# A re-examination of responding on ratio and regulated-probability interval schedules

**DOI:** 10.1016/j.lmot.2018.07.003

**Published:** 2018-11

**Authors:** Omar D. Pérez, Michael R.F. Aitken, Amy L. Milton, Anthony Dickinson

**Affiliations:** a Department of Psychology and Behavioural and Clinical Neuroscience Institute, University of Cambridge, Cambridge, UK; b Institute of Psychiatry, Psychology and Neuroscience, King’s College London, London, UK; c Nuffield College CESS-Santiago, Facultad de Administración y Economía, Universidad de Santiago de Chile, Santiago, Chile

**Keywords:** Reinforcement schedules, Dual-system theories, Ratio schedule, Interval schedule, Goal-directed behavior

## Abstract

The higher response rates observed on ratio than on matched interval reward schedules has been attributed to the differential reinforcement of longer inter-response times (IRTs) on the interval contingency. Some data, however, seem to contradict this hypothesis, showing that the difference is still observed when the role of IRT reinforcement is neutralized by using a regulated-probability interval schedule (RPI). Given the mixed evidence for these predictions, we re-examined this hypothesis by training three groups of rats to lever press under ratio, interval and RPI schedules across two phases while matching reward rates within triads. At the end of the first phase, the master ratio and RPI groups responded at similar rates. In the second phase, an interval group yoked to the same master ratio group of the first phase responded at a lower rate than the RPI group. Post-hoc analysis showed comparable reward rates for master and yoked schedules. The experienced response-outcome rate correlations were likewise similar and approached zero as training progressed. We discuss these results in terms of a contemporary dual-system model of instrumental conditioning.

## 1 Introduction

Two basic patterns of reward delivery are commonly used in instrumental conditioning experiments. In the first class, *ratio* schedules, reward delivery depends only on the number of responses performed, so that a reward is delivered every time a response requirement is attained. In the second class, *interval* schedules, the delivery of rewards depends not only on responding but also on time; a reward is scheduled to be delivered after an elapsed period of time, such that the first response performed after this time period is rewarded. The random-ratio (RR) and random-interval (RI) schedules are idealized stochastic versions of these schedules. The RR schedule sets a fixed reward probability per response, say q, so that the (1/q)th response is, on average, rewarded; the RI schedule programs the availability of a reward with a fixed probability, say r, in each second: rewards are therefore collected with probability 1 after an average of 1/r sec have elapsed.

An enduring debate in instrumental learning concerns the mechanisms driving performance under ratio and interval training. Ever since Thorndike (1911) espoused his Law of Effect, learning theorists have argued that probability of contiguous reward is the primary determinant of instrumental performance because response rates increase as the reward probability per response increases (e.g., see Belke et al., 2014; Mazur, 1983). However, the fact that ratio and interval performance differs even when the reward probability is matched (Catania et al., 1977; Dickinson, Nicholas, & Adams, 1983; Mazur, 1983; Peele, Casey, & Silberberg, 1984; Reed, 2001; Skinner, 2014; Zuriff, 1970) suggests that additional variables are involved in the instrumental learning process. Moreover, the ratio-interval difference persists when the reward rate (rather than reward probability) is matched, such that the reward probability experienced by interval-trained subjects is higher than the one experienced by those trained under the ratio schedule. This widely-observed result is problematic for theories of instrumental learning based on reward probability, both associative (Mackintosh & Dickinson, 1979; Mackintosh, 1974) and computational (Daw, Niv, & Dayan, 2005; Dezfouli & Balleine, 2012; Keramati, Dezfouli, & Piray, 2011; Niv, Daw, & Dayan, 2005).

This raises the questions as to what produces this difference. If the incentive value and the probability or rate of reward are the same, the expected value of both training regimes should be equal. So why does ratio training elicit more effort? To answer this question, probability-based theories shift the focus from individual responses to the pause between responses, or inter-response time (IRTs). On interval schedules, it is argued, longer IRTs yield higher reward probabilities for the next response that is to be emitted (Dawson & Dickinson, 1990; Peele et al., 1984; Tanno & Silberberg, 2012; Wearden & Clark, 1988). If the animal is sensitive to the differential reinforcement of longer IRTs, interval responding should hence be slowed compared to a ratio schedule under which reinforcement probability remains constant with variations in IRT. Even though the reward probability *per response* programmed by the experimenter might be similar, these theories stress the importance of the *joint* probability per response together with the time elapsed since the last response in order to explain responding (Niv, Daw, Joel, & Dayan, 2007; Peele et al., 1984; Shimp, 1973; Wearden & Clark, 1988).

One way of investigating the sufficiency of IRT-reinforcement in explaining instrumental performance is to design a schedule in which this factor is neutralized while keeping the average interval between rewards constant at the scheduled value. The regulated-probability interval schedule (RPI), originally designed by Kuch and Platt (1976), does this by continuously recording the local response rate and then setting the reward probability for the next response (*P*) to a value that maintains the scheduled reward rate if the animal continues to respond at the same rate. Thus, the reward probability for the next response is independent of the preceding IRT. Formally, if T is the scheduled interval between rewards, the RPI sets the reward probability for the *next* performed response to P=t/Tm, where t is the time it has taken the subject to perform the last *m* IRTs. Hence under the RPI schedule the reward probability is not determined entirely upon the current IRT, but on the duration of a number (m) of IRTs emitted before the current IRT. The RPI, in other words, considers a *local* response rate $B_{m}=(m+1)/t$ given by the last m + 1 responses (or m IRTs) in the last t secs, and fluctuates the reward probability inversely with respect to this local response rate so that the agent still receives a constant reward rate of 1/T rewards per second independently of the length of the current IRT. Since the current magnitude of IRT contributes only a fraction 1/m of the change in reward probability, the RPI should also be able to neutralize the effect of timing on increasing reward probabilities for long IRTs.

If IRT-reinforcement is sufficient to explain the difference between ratio and interval responding, response rates under RPI schedules should be higher than under RI training. And since IRT size is independent of reward probability in both ratio and RPI schedules, responding should be comparable between these two schedules.

The evidence for these predictions is, however, both mixed and scarce. In a within-subject study of lever pressing, Tanno and Sakagami (2008) trained four rats under ratio, RPI and interval schedules and found that ratio- and RPI-trained rats maintained comparable response rates. Consistent with an IRT-reinforcement hypothesis, they also observed lower responding under interval schedules than under both ratio and RPI schedules. However, a previous study of chain pulling by Dawson and Dickinson (1990) found higher responding on a ratio than on a RPI schedule yoking reward rates within three triads of rats, suggesting that an IRT-reinforcement mechanism is not sufficient to account for the whole ratio-interval difference. These two contradicting experiments and the fact that the majority of theories of instrumental conditioning have been informed by lever-pressing data, makes it important to further examine this hypothesis using this target response. This was the goal of the present experiment.

## 2 Experimental study

The present study comprised two phases of training and three groups of rats (see Table 1). In the first phase, the programmed reward rate for a RPI group was yoked to that generated by a master RR group. As IRT-reinforcement theory anticipates no difference between the performance of RR and RPI groups for matched reward rates, it was necessary to show that performance was also sensitive to reward probability. To this end, a third, RR/RI group received rewards with a higher probability than the master RR group and therefore should have responded at a higher rate. In the second phase, this group was switched to a standard RI schedule with the reward rate yoked to the same master RR group of the first phase. The contrast between RPI and RI performance in this second phase established whether responding was sensitive to the differential reinforcement of long IRTs by yielding a lower response rate in the RR/RI group.

**Table 1:** *Design of the Experiment.* The master group is written in bold. The subscript "y" signifies that the group was yoked to the master group with respect to reward rate.

		Group	
Phase	RR (N = 12)	RPI (N = 12)	RR/RI (N = 12)
1 (10 sessions)	**RR-20**	RPI-y	RR-10
2 (10 sessions)	**RR-20**	RPI-y	RI-y

### 2.1 Method

#### 2.1.1 Subjects

The subjects were 36 male naïve Lister Hooded rats (Charles River, Margate, UK) that were around 3 months old and with a mean free-feeding weight of 374 g at the beginning of the experiment. They were caged in groups of four in a vivarium under a 12-hour reversed light-dark cycle (lights off at 0700). All rats had ad libitum access to water in their home cages and were mildly food restricted throughout training by being fed for 1 h in their home cages after every session. This research was regulated under the Animals (Scientific Procedures) Act 1986 Amendment regulations 2012 under Project Licence 70/7548 following ethical review by the University of Cambridge Animal Welfare and Ethical Review Body (AWERB).

#### 2.1.2 Apparatus

Subjects were trained in twelve operant chambers (Med Associates, Vermont, USA) controlled by Whisker Server (Cardinal & Aitken, 2009). A client written in Visual Basic © and run on a laptop computer (ASUS © K52 J) under Microsoft Windows © 10 was utilized to communicate with the server, control the chambers and retrieve the data.

Each chamber had a magazine and two retractable levers at each side of the magazine. A pellet dispenser delivered 45-mg chocolate-flavored pellets (Sandown Scientific, Middlesex, UK) into the magazine. A 2.8-W house light illuminated the chamber during each experimental session.

#### 2.1.3 Behavioural procedures

##### 2.1.3.1 Pretraining

Rats were first given two magazine training sessions with both levers retracted. During these sessions, the pellets were delivered on a random time 60-sec schedule (i.e. a reward was programmed to be delivered on average after 60 s in the absence of the lever) until 30 rewards were delivered and consumed. On the next session, one of the levers was inserted at the start of the session and each press was rewarded on a fixed ratio (FR1) schedule. Finally, for each of the three subsequent days, lever pressing was rewarded under an RR schedule with increasing ratio requirements until 30 rewards were earned and consumed. The ratio parameter was set to 5 on Day 1, and 10 on Days 2 and 3. The active lever was counterbalanced between subjects, but each subject was trained with the same lever and in the same operant box throughout training. All rats were given two runs of pre-training until all rewards were delivered and consumed.

##### 2.1.3.2 Training

On the following day subjects were randomly assigned to 3 different groups (N = 12 each) (see Table 1) the first phase of the experiment, master rats in the RR group were run under an RR-20 schedule, so that 1 of every 20 responses on average was rewarded. On a second run, yoked subjects in the RPI group were trained on a RPI schedule (RPI-y) with the same inter-reward intervals produced by their corresponding master subjects in the RR group. On a third run, the RR/RI group was run under a RR-10 schedule, so that 1 of every 10 responses on average was rewarded.

In the second phase of the experiment, rats in the RR/RI group were switched to a RI schedule yoked to the master rats in the RR group in the same manner as rats in the RPI group, thereby yielding two interval groups yoked by reward rate within triads. Across all sessions of the experiment, the yoked rats were trained in the same operant chamber and with the same lever as their corresponding master rats. In order to minimize possible carry-over effects between phases - particularly in the RR/RI group which was shifted from a ratio to an interval schedule – all rats received at the end of each phase three sessions during which the food pellets were delivered non-contingently to lever-pressing on a random-time 60-sec schedule.

To prevent the development of extreme patterns of response bursting (Reed, 2011; Shull, Gaynor, & Grimes, 2001), for all schedules tested in the experiment the reward schedule was programmed so that only the first response in each 1-sec window interrogated the reward probability in the computer; all other responses during these windows were recorded but did not engage the probability generator. Although this effectively constrained the reward rate to an average of 3 rewards per min in the yoked RI and RPI groups, similar interval parameters have been used in previous studies reporting the ratio-interval difference for matched reward rates (see, for example, Dickinson et al., 1983). Additionally, to prevent rats from stopping responding when a reward had taken too many responses to be earned or too long an interval to be scheduled, we constrained these parameters to a maximum of three times the nominal response or interval requirement, respectively. For example, if the number of responses currently performed since the last reward in the RR-20 schedule was 60 responses, the next response was rewarded. Likewise, in the RPI or RI schedules, if the current interval since last reward was 3T, and T is the scheduled interval, then the next response was rewarded. Since Dawson and Dickinson (1990) found no evidence of memory size in the RPI schedule affecting performance, we set the value of m to 50. The same memory size was employed by Tanno and Sakagami (2008) and Dawson and Dickinson (1990) in their previous work.

Welch t-tests and Cohen’s D (with 95% confidence interval) were calculated for the pre-planned contrasts of interest. The significance of the contrasts was evaluated against the standard criterion of α=.05. When no statistical difference was found between groups, a Bayes Factor $\left(BF_{01}\right)$ in favor of the null was also calculated to test the likelihood of the null over the alternative hypothesis of there being a difference between the groups in terms of the variable being analyzed (Morey & Rouder, 2015). All the analyses were performed using the R programming language running in RStudio (RStudio Team, 2015) and extended with the packages BayesFactor, reshape2 v.1.4.1, plyr v.1.8.3, and ggplot2 v.2.1.0. Data and scripts for all the analyses can be found at https://doi.org/10.17863/CAM.22861.

### 2.2 Results

#### 2.2.1 Phase 1: an RR-10 schedule produced greater responding than an RR-20 schedule, with equivalent responding on the RPI-y schedule

**Fig. 1:**
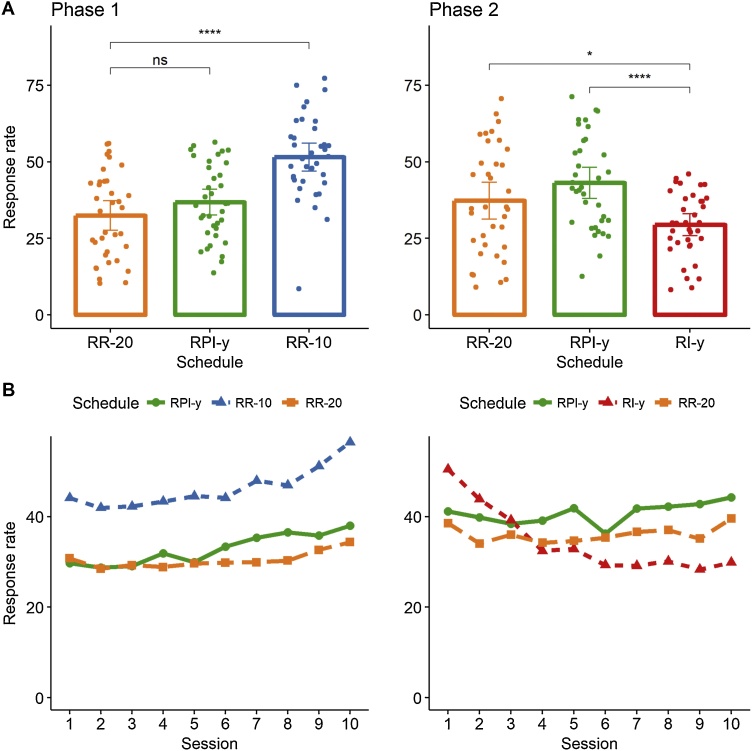
*Response rates for phases 1 and 2 of the experiment.***A**. Average response rates maintained by rats in the last 3 sessions of training in each phase of the study. **B**. Average response rates across the 10 sessions of training in each phase of the study. * p < .05, ****p < .001, ns: not significant. Error bars represent 95% bootstrapped confidence intervals.

Fig. 1A presents the response rates in the last three sessions of training for each group and phase of the study; the acquisition curves across all sessions are presented in Fig. 1B. The results from Phase 1 supported the prediction of all instrumental theories with regard to RR schedules with different reward probabilities in that rats trained under the RR-10 schedule responded more vigorously than those trained under the RR-20 schedule, $t(69.9)=-5.82,p<.01,D=-1.37\;\;95\%\;CI\left[-1.90,-0.85\right]$. In contrast, there was no detectable difference in responding between rats trained under RR-20 and those trained under the RPI-y schedule at the end of the first phase of training, $t(68.7)=-1.39,p=.17,D=-0.33\;\;95\%\;CI\left[-0.80,0.14\right]$.

#### 2.2.2 Phase 2: responding of an RI-y schedule was lower than both responding on an RR-20 schedule and responding on an RPI-y schedule

To test the effect of differential IRT reinforcement on responding, in Phase 2 the group RR/RI was switched to an RI-y schedule yoked to the master RR group with respect to reward rate in the same way as the RPI group was yoked to the RR group in both phases. The RR group responded at a higher rate than the RI group, $t(62.8)=4.44,p<.01,D=0.24\;95\%\;CI\left[-0.23,0.72\right]$, replicating the widely-observed ratio-interval difference when regular interval schedules are employed (Dickinson et al., 1983; Peele et al., 1984). Importantly, the RI group also responded at a lower rate than the RPI group, t(57.1)=2.27,p=03,D=0.57 95% CI[0.09,1.05], showing that the differential reinforcement for long IRTs was able to slow responding in the RI group compared to a reward that does not hold this property.

#### 2.2.3 Yoking analysis

The yoking analysis was performed separately for the last 3 sessions of each phase of the study. For each phase, possible differences between those variables that were intended to be matched by the yoking procedure were analyzed; in particular, the interest was put in ensuring the yoking was successful in matching reward rates and, in addition, whether the RPI schedule was successful in controlling the differential reinforcement of long IRTs. Table 2 shows the results for each phase of the experiment.

**Table 2:** *Results of the yoking procedure.* Mean and 95% bootstrapped confidence intervals for the last three sessions of phases 1 and 2 of the study. The reward rates are in rewards per min; the IRT difference is calculated as the difference between the mean reinforced IRT and the mean IRT emitted by each rat in seconds. The letter “y” signifies that the group was yoked to the reward rate obtained in the master ratio group.

Phase	Variable	Schedule		
		RR-20 (master)	RPI-y	RR-10
1	Rewards per minute	1.32 [1.16, 1.48]	1.46 [1.27, 1.64]	3.64 [3.34, 3.94]
	IRT difference (sec)	−0.52 [−9.67, 8.63]	−1.77 [−2.26, -1.28]	−6.52 [−7.67, −5.37]

		RR-20 (master)	RPI-y	RI-y
2	Rewards per minute	1.79 [1.53, 2.05]	2.08 [1.76, 2.38]	1.62 [1.39, 1.85]
	IRT difference (sec)	−14.89 [−18.43, −11.35]	−16.53 [−19.77, −13.29]	3.31 [2.45, 4.17]

#### 2.2.4 Reward rate

The reward rates obtained in each session were similarly analyzed for the last 3 sessions of the two phases of the experiment (Table 2). The results confirmed that the yoking procedure succeeded in matching the reward rates with respect to the master RR 20 group (Phase 1: RR-20 - RPI-y: $t(68.8)=-1.15,p=.25,D=-0.27\;95\%\;\;CI\;[-0.74,0.20],BF_{01}=2.34$; Phase 2: RR-20 - RPI-y: $t(67.9)=-1.42,p=.16,D=-0.33\;95\%\;CI\;[-0.81,0.14],BF_{01}=1.74$; RR-20-RI-y: $t(68.8)=1.03,p=.30,D=0.24\;95\%\;CI\;[-0.23,0.72],BF_{01}=2.60$.)

#### 2.2.5 IRT reinforcement

Unlike RI schedules, the RPI schedule aims not to assign responses followed by longer IRTs a higher reward probability. The same property holds for the RR schedule, although for a simpler reason: in this case, the probability of obtaining a reward depends only on each response independently, and not on the time that has elapsed since the last reward obtained. Therefore, the difference between the mean rewarded IRT size and mean emitted IRT size is an index of whether the reward schedule was rewarding responses followed by long IRTs over all other emitted IRTs. The index, therefore, should be less than or equal to zero in RR and RPI schedules. In contrast, since the reward probability is a direct function of IRT size on the RI schedule, the value of this index for RI schedules should be positive. If both predictions are confirmed by the data, then the differences between the RPI and RI groups in the second phase of the study can be attributable to the latter schedule rewarding long pauses between responses in a higher proportion. The data (Table 2) confirmed that this was the case, as only the RI- y schedule yielded a positive IRT-difference index. In addition, the index was lower for both RR-20 and RPI-y schedules at the end of the second phase compared to the end of the first phase. This may be a consequence of the development of response bursting with extended training, meaning that reinforcement was more likely to follow a series of short IRTs than a series of long IRTs (see Reed, 2011, 2015; Shull, 2011).

## 3 Discussion

Using lever pressing in rats, the present study re-examined the predictions of IRT-reinforcement theories regarding ratio and interval performance under matched reward rates. The first phase confirmed the critical role of reward probability in instrumental responding, as indicated by the higher response rates observed under RR-10 training compared to RR-20 training. By contrast, there was no detectable difference in responding between the RR group and the yoked RPI group. The second phase of the experiment confirmed the widely observed ratio-interval difference, in that the RR group came to perform at a higher rate than the yoked RI group (1999, Cole, 1994; Peele et al., 1984; Zuriff, 1970) at the end of training. Direct evidence for the role of differential reinforcement of long IRTs was revealed in the lower responding on the RI group compared to the RPI group in this second phase. These data agree with those reported by Tanno and Sakagami (2008), who found similar rates of lever-pressing in RR and RPI schedules and higher responding on these two schedules compared to a RI schedule using within-subject yoking of reward rates. By contrast, our data are at variance with those reported by Dawson and Dickinson (1990), who found higher rates of chain-pulling on RPI than on RI schedules, and lower rates on RPI than RR schedules using between-subject yoking of reward rates within triads.

At issue is why Dawson and Dickinson (1990) obtained higher ratio than RPI performance in their chain-pulling study. Based on a previous idea originally espoused by Baum (1973), they speculated that this difference may have been a consequence of the different linear response-reward rate correlation (r-c) that subjects experience under these two schedules. Under ratio training, changes in response rate are followed by changes in reward rate. By contrast, the reward rate is fixed at the reciprocal of the programmed inter-reward interval [(1/q) - sec] on interval schedules. Assuming that performance is directly related not only to reward rate but also to the experienced r-cs, ratio training should sustain higher response rates than RPI training.

Dickinson and Pérez (2018) have recently offered a dual-system model of instrumental learning that includes a specific computational rule for calculating this experienced r-c. In their model, the experienced r-c is computed by dividing subject’s working-memory in a number of time-samples and counting the number of responses and rewards in each of these samples. A correlation coefficient can thus be computed as a measure of this experienced response-reward r-c. In this model, subjects will experience positive r-cs only under two conditions. First, responding needs to vary sufficiently across time-samples in memory so that subjects are able to experience that changes in their response rate are followed by changes in reward rate; stable or high response rates narrow the reward and response rates sampled and decreases the experienced r-c (Dickinson & Pérez, 2018). Thus, one possibility is that chain-pulling in Dawson and Dickinson’s (1990) experiment was more variable than lever pressing in both the present and Tanno and Sakagami’s (2008) studies. For example, the mechanical properties (e.g. degree of hysteresis) of the microswitch from which the chains were suspended (a home-made system) may have contributed to a sustained variation in responding, allowing the rats to constantly experience positive r-cs. The second condition is that the ratio parameter under which the rats are trained is sufficiently small so as to establish positive experienced r-cs. In this regard, the low reward probability set by the RR-20 schedule employed in the present study may not have been sufficient to establish positive r-cs. If behavior is jointly controlled by experienced r-c and reward rate, (Dickinson & Pérez, 2018) and this latter variable is equated by the yoking procedure, it follows that RR and RPI schedules should support comparable response rates, as observed in this study.

**Fig. 2:**
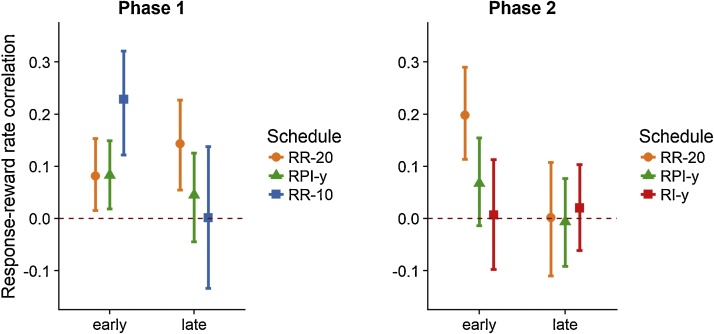
*Response-reward rate correlations experienced by each group in Phase 1 and Phase 2 of the experiment.* The values shown are mean and 95% bootstrapped confidence intervals for the first (early) and last (late) three sessions of training for each rat and group. (see Appendix for details).

Following Dickinson and Pérez’s (2018) approach, we explored these possibilities by calculating the r-cs experienced by subjects early and late in training in each phase of our study (see Appendix for details). Fig. 2 presents the average r-cs experienced by rats in Phase 1 (left panel) and Phase 2 (right panel) of the study. Given that rats came to perform at a high and stable rate under RR-10 training, the r-c experienced by this group converged to zero rapidly, as indicated by the confidence interval including both positive and negative values. However, consistent with the idea that the variation of responding across samples should establish positive experienced r-cs for limited training, we found that rats experienced higher r-cs early in training under RR-10 than RR-20 training (t(58.6)=2.39,p=.02). The RR and RPI groups, on the other hand, experienced positive r-cs during the first phase, but these did not differ either early(t(63)=0.44,p=.66) or late(t(58.3)=1.49,p=.14) in training. In the second phase, the effect of stable responding on experienced r-cs was evident in that the r-cs approached zero in all groups at the end of training (confidence intervals including both positive and negative values). In addition, there was no difference between the r-cs experienced at the end of training across the three groups (F(2,104)=0.90,p=.41). These results indicate that the choice of the RR-20 schedule was not able to sustain higher r-cs than the RPI-y schedule (t(65.8)=0.11,p=.91), explaining why we failed to detect any differences between RR and RPI groups.

Evidence for a role of experienced r-c in schedule performance has been presented by Reed (2006). Yoking reward rates, he compared performance on the RI-plus-linear feedback schedule (RI+) (McDowell & Wixted, 1986; Reed, 2007a, 2007b; Soto, McDowell, & Dallery, 2006) to that of a ratio schedule. Under the RI + schedule, higher reward probabilities for long IRTs are established in conjunction with positive response-reward r-cs. Reed reported evidence of an RI + schedule sustaining similar lever-pressing rates to an RR schedule, suggesting that the response-reward r-c was more important than IRT-reinforcement in increasing responding. Importantly, this observation held only when he encouraged his rats to respond at a sufficiently low rate by increasing the force required to depress the lever. When high response rates were encouraged by decreasing the required force, rats responded at a higher rate in the RR than in the RI+, suggesting that r-c was not driving performance under high response rates. The high response rates attained may have decreased the experienced r-cs, leaving reward rate and IRT-reinforcement as the main variables controlling responding.

In contrast with our observation of similar r-cs for RR and RPI schedules, Tanno and Sakagami (2008)’s reported higher r-cs on RR than RPI and RI schedules. Two reasons may explain this discrepancy. First, our method is based on a particular view of the computation that the subjects perform during training (Dickinson & Pérez, 2018). Rather than taking the overall response and reward rates per session and comparing across sessions as Tanno and Sakagami (2008) did, we have adopted an approach in which subjects take different time-samples and compute the experienced r-c within a session deploying a limited number of these samples. Thus, it is not the objective response-reward contingency or *feedback function* (Baum, 1973, 1992; Nevin & Baum, 1980; Soto et al., 2006) what we are interested in exploring in this paper, but rather the r-c that each subject *experiences* within and across sessions. Our analysis has shown that the RR-20 was not able to produce higher r-cs than interval schedules for matched reward rates. This is especially so late in training, when responding stabilises and the r-cs tend to decrease as a consequence of the little variation across memory samples.

Although the conditions that allow subjects to experience higher r-cs on ratio than interval schedules have yet to be systematically explored, the results reported here have shed light into this question and confirmed the predictions of IRT-reinforcement theory with respect to the ratio-interval contrast: if the variation of responding or the ratio parameter are not sufficient to establish higher r-cs for ratio than yoked RPI schedules, responding is driven only by reward rate and performance on ratio schedules is comparable to that of RPI schedules; compared to RPI schedules, responding under RI schedules is slowed by the differential reinforcement of long IRTs.
