# Supplementary material for: A re-examination of responding on ratio and regulated-probability interval schedules
Source: Learn Motiv. 2018 Nov;64:1–8. doi: 10.1016/j.lmot.2018.07.003 (PMC6264404; doi:10.1016/j.lmot.2018.07.003)
Supplement: Supplementary file 1 [file mmc1.docx]

**Appendix**

**A.1. Method for calculating response-reward rate correlation.**

Each session of training was divided in 60-sec time samples. If the session length was not a multiple of 60, the last time-sample was discarded from the data. For each session and rat, the response-reward r-c was calculated by taking the number of responses $(n_{resp}^{(j)})$ and rewards $(n_{rew}^{(j)})$ in each time-sample$j$ and calculating a Pearson correlation coefficient as$\frac{1}{K}\sum_{j=1}^{K} \frac{(n_{resp}^{(j)}-\overline{n_{resp}})(n_{rew}^{(j)}-\overline{n_{rew}})}{\sigma_{resp} \sigma_{rew}}$, where $\overline{n_{resp}}$and $\overline{n_{rew}}$are the mean number of responses and rewards in the session, respectively, and $\sigma_{resp}$ and $\sigma_{rew}$ their corresponding standard deviations. $K$ is the number of time-samples in each session. The values shown in Fig. 2 are the average of the first (*early*) or last 3 (*late*) sessions of training obtained for each rat. Since Pérez (2017) showed that the size of the time sample chosen does not exert an impact in the r-cs provided responses rates are sufficiently high, the value of 60-sec was chosen arbitrarily for this calculation.

**REFERENCES**

Pérez O.D., A cooperative dual-system model of instrumental conditioning. PhD Thesis. Cambridge University, Cambridge, England.
